# Cembranoid-Related Metabolites and Biological Activities from the Soft Coral *Sinularia flexibilis*

**DOI:** 10.3390/md16080278

**Published:** 2018-08-09

**Authors:** Chia-Hua Wu, Chih-Hua Chao, Tzu-Zin Huang, Chiung-Yao Huang, Tsong-Long Hwang, Chang-Feng Dai, Jyh-Horng Sheu

**Affiliations:** 1 Department of Marine Biotechnology and Resources, National Sun Yat-sen University, Kaohsiung 804, Taiwan; cathywu7979@gmail.com (C.-H.W.); slime112229@gmail.com (T.-Z.H.); huangcy@mail.nsysu.edu.tw (C.-Y.H.); 2 School of Pharmacy, China Medical University, Taichung 404, Taiwan; chchao@mail.cmu.edu.tw; 3 Chinese Medicine Research and Development Center, China Medical University Hospital, Taichung 404, Taiwan; 4 Graduate Institute of Natural Products, College of Medicine, and Chinese Herbal Medicine Research Team, Healthy Aging Research Center, Chang Gung University, Taoyuan 333, Taiwan; htl@mail.cgu.edu.tw; 5 Research Center for Chinese Herbal Medicine, Research Center for Food and Cosmetic Safety, and Graduate Institute of Health Industry Technology, College of Human Ecology, Chang Gung University of Science and Technology, Taoyuan 333, Taiwan; 6 Department of Anesthesiology, Chang Gung Memorial Hospital, Taoyuan 333, Taiwan; 7 Institute of Oceanography, National Taiwan University, Taipei 112, Taiwan; corallab@ntu.edu.tw; 8 Institute of Natural Products, Kaohsiung Medical University, Kaohsiung 807, Taiwan; 9 Department of Medical Research, China Medical University Hospital, China Medical University, Taichung 404, Taiwan; 10 Frontier Center for Ocean Science and Technology, National Sun Yat-sen University, Kaohsiung 804, Taiwan

**Keywords:** cembranoid-related compounds, flexibilisin, secoflexibilisolide, flexibilisolide, *Sinularia flexibilis*

## Abstract

Five new cembranoid-related diterpenoids, namely, flexibilisins D and E (**1** and **2**), secoflexibilisolides A and B (**3** and **4**), and flexibilisolide H (**5**), along with nine known compounds (**6**–**14**), were isolated from the soft coral *Sinularia flexibilis.* Their structures were established by extensive spectral analysis. Compound **3** possesses an unusual skeleton that could be biogenetically derived from cembranoids. The cytotoxicity and anti-inflammatory activities of the isolates were investigated, and the results showed that dehydrosinulariolide (**7**) and 11-*epi*-sinulariolide acetate (**8**) exhibited cytotoxicity toward a limited panel of cancer cell lines and 14-deoxycrassin (**9**) displayed anti-inflammatory activity by inhibition of superoxide anion generation and elastase release in *N*-formyl-methionyl-leucyl-phenylalanine/cytochalasin B (fMLF/CB)-induced human neutrophils.

## 1. Introduction

**Figure 1:**
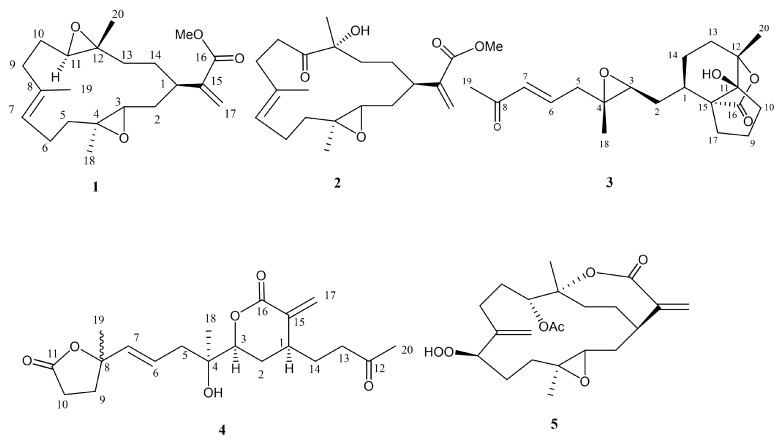
Structures of **1**–**5**.

**Figure 2:**
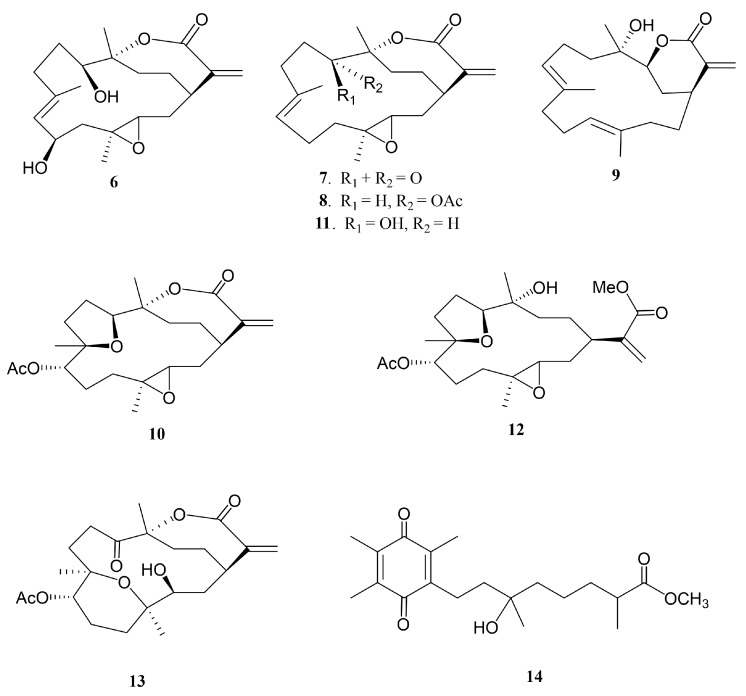
Structures of metabolites **6**−**14**.

Soft corals have been known to be the organisms possessing secondary metabolites with high diversity in chemical structures [1]. Since 1975, many cembranoid-type natural products with diverse and important biological activities have been isolated from *Sinularia flexibilis* [2,3,4,5,6,7,8,9,10,11,12,13]. In previous studies of the chemical constituents of Taiwanese soft corals, numerous marine metabolites with cytotoxic [14,15], neuroprotective [16], and anti-inflammatory activities [17,18,19] were also found from *Sinularia flexibilis*. Some cembranolides, possessing an α-methylene lactone ring, have been discovered as potent cytotoxic agents to a limited panel of cancer cell lines, for example, 14-deoxycrassin [20], and as significant anti-inflammatory agents, for example, sinulariolone acetate [21]. Additionally, cembranoids and related compounds from other coral species were also found to have notable antiviral [22], anti-inflammatory [23,24], and antiproliferative [25,26,27,28] activities. A wide variety of chemical diversity and biological activity of cembranoids encouraged us to search for more natural products from soft coral *S*. *flexibilis*, collected off the waters of Taiwan. Herein, we report the isolation of five new cembrane-related metabolites, namely, flexbilisins D and E (**1** and **2**), secoflexibilisolides A and B (**3** and **4**), and a cembranolide flexibilisolide H (**5**) (Figure 1), and nine known compounds, including 6*R*-hydroxysinulariolide (**6**) [3], 11-dehydrosinulariolide (**7**) [18], 11-*epi*-sinulariolide acetate (**8**) [13], 14-deoxycrassin (**9**) [20], 3,4:8,11-bisepoxy-7-acetoxycembra-15(17)-en-1,12-olide (**10**) [6], sinulariolide (**11**) [2], sinulaflexiolide E (**12**) [10], querciformolide A (**13**) [21], and flexibilisquinone (**14**) [19] (Figure 2). Their structures were established by spectroscopic analysis including infrared (IR), mass spectrometry (MS), and nuclear magnetic resonance (NMR) data (Figures S1–S14), as well as chemical transformation. The biogenetic origins of **3** and **4** were postulated to demonstrate the relationship between stereochemistry and biogenetic implications. The cytotoxicity of the isolates toward a limited panel of cancer cell lines and their inhibition of superoxide anion generation and elastase release in *N*-formyl-methionyl-leucyl-phenylalanine/cytochalasin B (fMLF/CB)-induced human neutrophils were also investigated.

## 2. Results and Discussion

**Figure 3:**
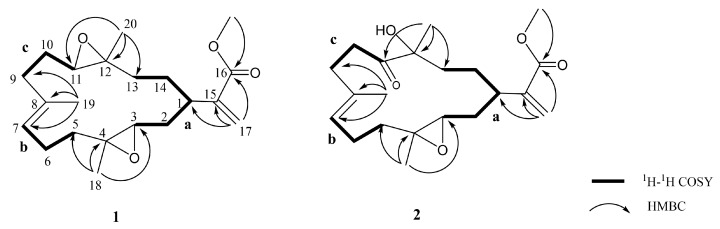
Key COSY and HMBC correlations for **1** and **2**.

Flexbilisin D (**1**) was obtained as a colorless oil and its high-resolution electrospray ionization mass spectrometry (HRESIMS) (*m/z* 371.2196 [M + Na]^+^) data established the molecular formula of C_21_H_32_O_4_, indicating six degrees of unsaturation. The IR spectrum showed the absorption bands of carbonyl group (1716 cm^−1^) and double bond (1647 cm^−1^). The NMR data (Table 1), including ^13^C NMR and distortion less enhancement by polarization transfer (DEPT) spectra, displayed 21 carbin signals which can be classified as three methyls (*δ*c 15.3, 17.0, 17.8), one methoxy (*δ*c 52.0), seven *sp*^3^ methylene (*δ*c 22.2, 24.6, 27.4, 31.5, 33.0, 36.0, 36.9), one *sp*^2^ methylene (*δ*c 124.4), three *sp*^3^ methine (*δ*c 35.2, 60.1, 61.9), one *sp*^2^ methine (*δ*c 126.5), two *sp*^3^ quaternary (*δ*c 60.2, 60.8), and three *sp*^2^ quaternary (134.2, 142.7, 167.3) carbons. The ^13^C NMR signals at *δ*c 142.7 (C), *δ*c 124.4 (CH_2_) and the ^1^H NMR signals at *δ*_H_ 6.26 (1H, s) and *δ*_H_ 5.48 (1H, s) revealed the presence of a 1,1-disubstituted double bond, while those at *δ*c 134.2 (CH), *δ*c 126.5 (C), and *δ*_H_ 5.18 (1H, t, *J* = 5.6 Hz) are indicative of a trisubstituted double bond. Two trisubstituted epoxides were identified from the NMR signals at *δ*c 61.9 CH, 60.8 C and 60.2 C, 60.1 CH; *δ*_H_ 2.65 dd, *J* = 10.0 and 3.2 Hz; 2.81 dd, *J* = 10.0 and 4.0 Hz. The molecular skeleton of **1** was established by the above results, as well as the correlations spectroscopy (COSY) and heteronuclear multiple bond correlation (HMBC) correlations as shown in Figure 3. By analysis of the COSY correlations, it was possible to identify three partial structures (a–c). The fact that the methyl ester group [CO (*δ*c 167.3)/OMe (*δ*c 52.0; *δ*_H_ 3.76)] was on C-15 (*δ*c 142.7) was confirmed by the HMBC correlation from H_2_-17 (*δ*_H_ 6.26, 5.48) to C-16 (*δ*c 167.3). The two epoxides were assigned at 3,4- and 11,12-positions with methyl substituents at C-4 (*δ*c 60.2) and C-12 (*δ*c 60.8) according to HMBC correlations from H_3_-18 (*δ*_H_ 1.29) to C-3 (*δ*c 60.1), C-4, and C-5 (*δ*c 36.9), as well as H_3_-20 (*δ*_H_ 1.23) to C-11(*δ*c 61.9), C-12, and C-13 (*δ*c 33.0), respectively. Together with other key HMBC correlations from H_3_-19 (*δ*_H_ 1.65) to C-7 (*δ*c 126.5), C-8 (*δ*c 134.2), and C-9 (*δ*c 36.0), as well as H_2_-17 to C-1 (*δ*c 35.2), C-15, and C-16 permitted the establishment of the carbon skeleton.

**Figure 4:**
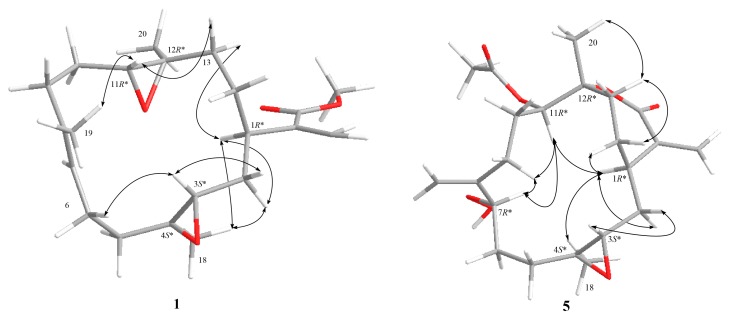
Selective NOESY correlations for **1** and **5**.

The relative configuration of **1** was determined by the nuclear Overhauser effect spectroscopy (NOESY) experiment as shown in Figure 4. Assuming that H-1 is α-oriented, which showed NOESY correlations to one of H_2_-2 (*δ*_H_ 1.36) and one of H_2_-13 (*δ*_H_ 1.70); thus, another of H_2_-2 (*δ*_H_ 2.05) and H_2_-13 (*δ*_H_ 1.20) were β-oriented. Two *trans* epoxides were then assigned according to the NOESY correlations from H-11 to H-13*β* and from H-13α to H_3_-20, as well as those from H_3_-18 to H-2α. The proposed structure of **1** is in agreement with the most stable conformation (Figure 4) generated by an energy-minimized (MM2) force field calculation [29]. Consequently, the relative configurations of C-1, C-3, C-11, and C-12 were determined as 1R*, 3S*, 4S*, 11R*, 12R* (Figure 4).

Flexibilisin E (**2**) had a molecular formula C_21_H_32_O_5_ as deduced from HRESIMS data. The ^1^H and ^13^C NMR spectra showed that the structure of **2** closely resembled that of **1**. By comparing their NMR data (Table 1), significant differences in chemical shifts were observed at C-11 (*δ*c 61.9 for **1**; 213.6 for **2**), C-12 (*δ*c 60.8 for **1**; 78.8 for **2**), and C-20 (*δ*c 17.0 for **1**; 25.7 for **2**), suggesting that **2** is a 12-hydroxy-11-oxo derivative of **1**. This was supported by the COSY and HMBC correlations (Figure 3). The structure and absolute configurations of the stereogenic centers of **2** were confirmed by an alkaline hydrolysis of 11-dehydrosinulariolide (**7**), whose absolute configurations at C-1, C-3, C-4, and C-12 were determined as 1*R*, 3*S*, 4*S*, and 12*R*, respectively, based on a single-crystal X-ray diffraction analysis [18]. Accordingly, the structure of **2** was determined as shown in Figure 1.

**Figure 5:**
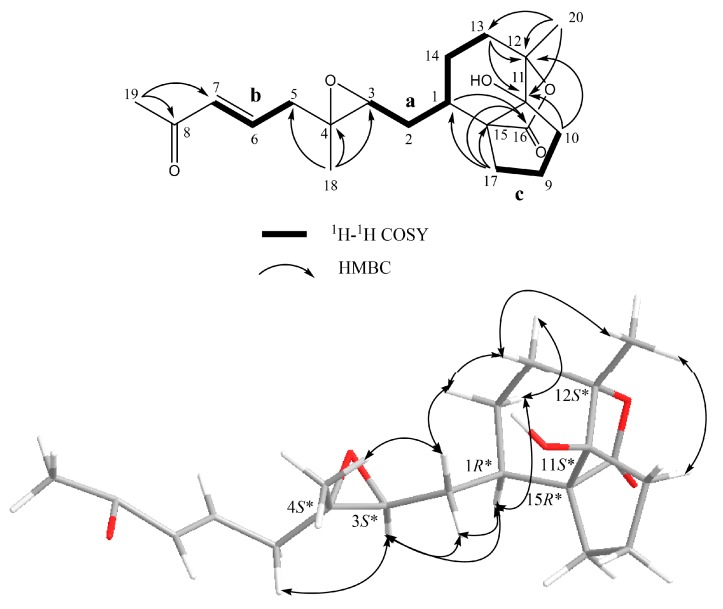
Key COSY and HMBC correlations and selective NOESY correlations for **3**.

Secoflexibilisolide A (**3**) was isolated as a colorless oil and its molecular formula was established as C_20_H_28_O_5_ by the observation of a sodiated molecular ion peak at *m/z* 371.1831 (calcd. for 371.1829 [M + Na]^+^) in the HRESIMS, indicating seven degrees of unsaturation. Its IR absorption bands suggested the presence of hydroxy (3450 cm^−1^) and carbonyl (1765 and 1671 cm^−1^) groups. Three spin systems (a–c), inferred by analysis of the COSY correlations, were constructed as shown in Figure 5. A bicyclo[4.3.0]nonane ring system was established by the HMBC correlations from H_3_-20 (*δ*_H_ 1.39) to C-11 (*δ*c 85.3), C-12 (*δ*c 84.7), and C-13 (*δ*c 30.4); from H_2_-10 (*δ*_H_ 2.05, 1.68) to C-11 and C-12; and from H_2_-17 (*δ*_H_ 2.26, 1.66) to C-1 (*δ*c 34.5), C-15 (*δ*c 60.9), and C-16 (*δ*c 177.6). In addition, a γ–lactone ring between C-16 and C-11 was evidenced by the IR absorption band at 1765 cm^−1^, which is consistent with perhydroindan analogues [30]. A methyl-substituted epoxy group was evidenced by the HMBC correlations from H_3_-18 (*δ*_H_ 1.28) to C-3 (*δ*c 61.9), C-4 (*δ*c 58.8), and C-5 (*δ*c 41.2), while an acetyl group was found to be located at C-7 by the HMBC correlations from H_3_-19 (*δ*_H_ 2.26) to C-7 (*δ*c 134.0) and C-8 (*δ*c 198.3). Accordingly, the planar structure of **3** was deduced as shown in Figure 5.

**Scheme 1:**
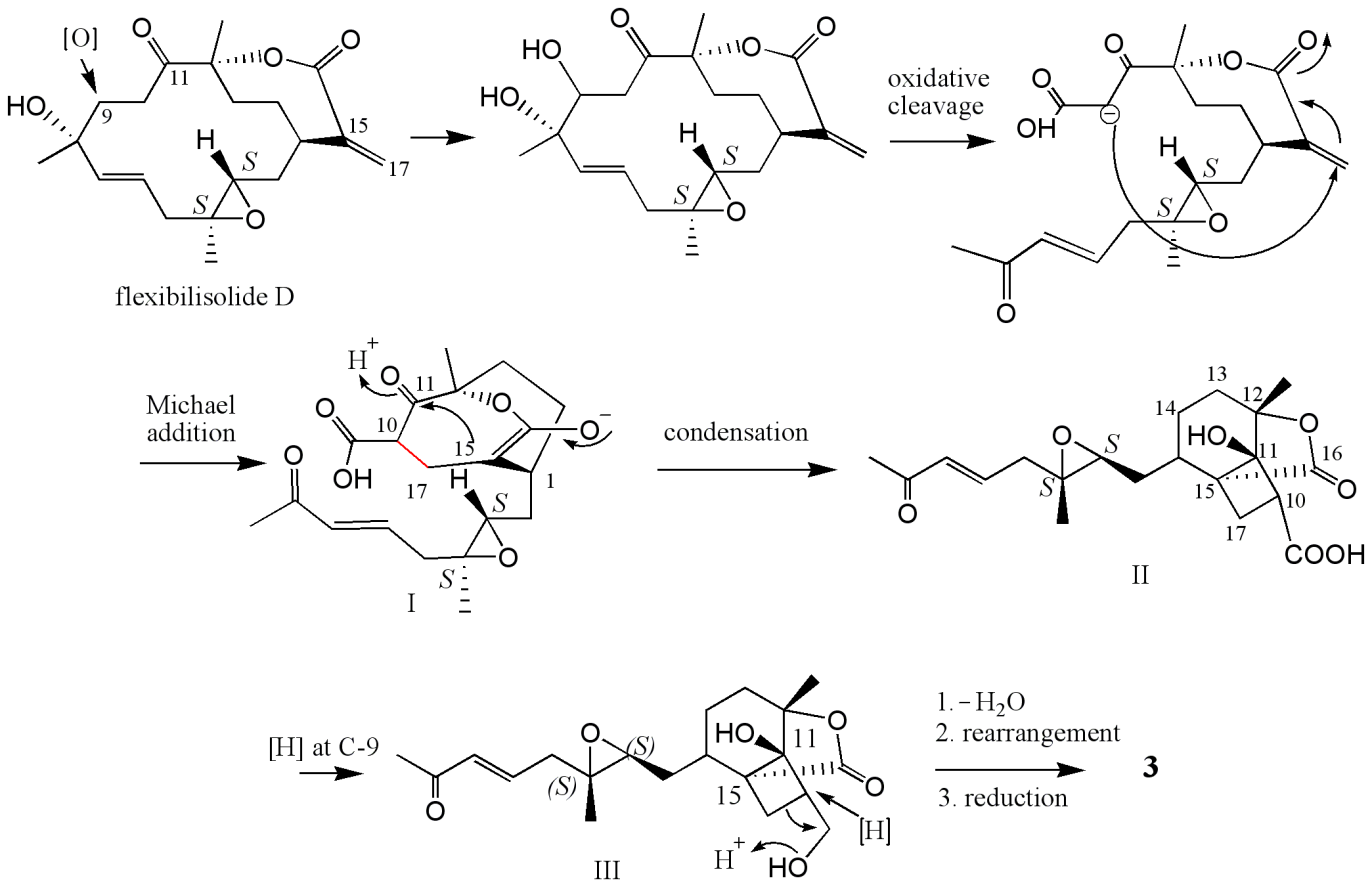
Proposed biosynthetic pathway for **3**.

Compound **3** can be hypothesized to derive from a precursor with the cembranoid-type skeleton. As shown in Scheme 1, flexibilisolide D, which was also isolated from this coral [17], was suggested as a precursor. Flexibilisolide D was converted to intermediate I by oxidative cleavage and Michael addition. Reduction of the carboxylic acid of the cyclobutane intermediate (II), derived from I by the aldol condensation, would produce an alcohol functionality in III. Rearrangement of the hydroxymethyl cyclobutane through a reductive ring opening in III resulted in a formation of the cyclopentane ring in **3**. This suggested that the configurations of C-1, C-3, C-4, and C-12 should be the same as those of flexibilisolide D and related analogues possessing a 3,4-epoxide group, isolated previously from this soft coral [17]. The relative configurations of C-11 and C-15 were determined by a combination of NOESY correlations (Figure 5) and pyridine-induced solvent shift experiment [31]. The NOESY correlations from H_3_-20 to H_2_-10 allowed the assignment of the lactone ring as α-oriented. However, the present NOESY data were unable to fully confirm the orientation of OH-11. As a result, the pyridine-induced solvent shift experiment was applied on **3**. H-2β (*δ*_H_ 1.66 in pyridine), which is 1,3-diaxial to OH-11, was found to be downfield shifted by 0.28 ppm with respect to H-2β, measured in CDCl_3_ (Table 2), suggesting the β-orientation of OH-11. Consequently, the relative configurations 1R*, 3S*, 4S*, 11S*, 12S*, 15R* were suggested for **3**.

**Figure 6:**
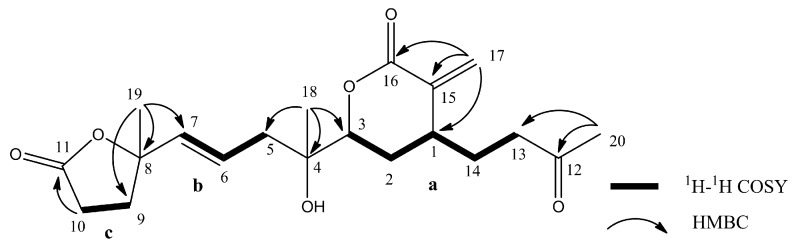
Key COSY and HMBC correlations for **4**.

The molecular formula of **4** was found to be C_20_H_28_O_6_ as deduced from the HRESIMS and ^13^C NMR data, suggesting seven degrees of unsaturation. The ^13^C NMR data of **4** (Table 3) showed signals attributable to two double bonds (*δ*c 139.5, 126.0, 136.9, 124.1), one ketone carbonyl (*δ*c 205.6), and two ester carbonyls (*δ*c 175.4, 165.3), which accounted for five degrees of unsaturation. Accordingly, the structure of **4** may contain two rings. From the COSY spectrum of **4**, it was possible to suggest the presence of three proton sequences for H_2_-13/H_2_-14/H-1/H-2/H-3, H_2_-5/H-6/H-7, and H_2_-9/H_2_-10 (a–c, respectively; Figure 6). Key HMBC correlations from H_3_-20 (*δ*_H_ 1.64) to C-12 (*δ*c 205.6) and C-13 (*δ*c 39.4); from H_2_-17 (*δ*_H_ 6.40, 5.12) to C-15 (*δ*c 139.5), C-16 (*δ*c 165.3), and C-1 (*δ*c 35.9); from H_3_-18 (*δ*_H_ 1.00) to C-3 (*δ*c 83.0), C-4 (*δ*c 72.5), and C-5 (*δ*c 41.3); and from H_3_-19 (*δ*_H_ 1.04) to C-7 (*δ*c 136.9), C-8 (*δ*c 83.9), and C-9 (*δ*c 33.9); as well as H_2_-10 (*δ*_H_ 2.05, 1.94) to C-11 (*δ*c 175.4) were observed, allowing to establish the planar structure of **4** as shown in Figure 6.

**Scheme 2:**
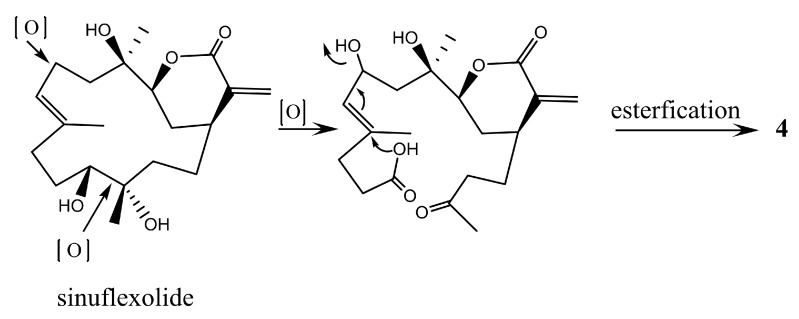
Proposed biosynthetic pathway for **4**.

In the NOESY spectrum of **4**, correlations from H-2α to both H-1 and H-3, and from H_3_-18 to H-3 revealed that H-1 and H-3 are on the same face of the lactone ring. Biogenetically, **4** could be derived from sinuflexolide, which has been reported from the same coral [12], via an oxidative cleavage of the diol groups, epimerization of the vinyl alcohol, and subsequent esterification (Scheme 2). This suggested that the configuration of C-4 should be the same as that of sinuflexolide. Consequently, the relative configurations 1S*, 3S*, 4R* were suggested for **4**. The configuration of C-8 remains undetermined.

The ^13^C NMR data (Table 3) and the HRESIMS of **5** showed that it has the same molecular formula as flexibilisolide G [17], that is, C_22_H_32_O_7_. They also have similar functional groups, including an α-exomethylene-ε-lactone ring, an acetoxyl group, and a methyl-substituted epoxy group. However, the obvious difference between the two compounds is that the 6,7-double bond in flexibilisolide G is isomerized to an exocyclic double bond at C-8 in **5**. In addition, the hydroperoxy group at C-7 in flexibilisolide G was found to migrate to C-8 in **5**. The above findings were further confirmed by COSY and HMBC correlations. The 1R*, 12R* configuration of the α-exomethylene-ε-lactone ring was deduced based on the NOESY correlations from H-1 to H-14α, from H_3_-20 to H-13β, and from H-14β to H-13β. In addition, NOESY correlations from H-11 to both H-7 and H-1 as well as H-1 to H-4 suggested the relative configurations of C-3, C-4, C-7, and C-11 as shown in Figure 4.

The cytotoxicitiy of **1**, **2**, and **4**–**14** against P-388 (murine leukemia), K-562 (human erythromyeloblastoid leukemia), and HT-29 (human colon carcinoma cells) cell lines was investigated. The results showed that **7**–**9** and **11** exhibited cytotoxic activity toward P-388 and K-562 cancer cell lines with half maximal inhibitory concentration (IC_50_) values ranging from 6.9 μM to 26.7 μM (Table 4). Among them, **7** showed selective cytotoxicity toward P-388, while **8** was found to show potent activity and selectivity toward P-388 and HT-29 cancer cell lines. Compounds **1**, **2**, **4**–**6**, **10**, and **12**–**14** were nontoxic toward these cancer cell lines (IC_50_ values > 40 μM). The anti-inflammatory effect of **1**, **2**, and **4**–**14** was also studied by measuring their ability to suppress *N*-formyl-methionyl-leucyl-phenylalanine/cytochalasin B (fMLF-CB)-induced superoxide anion (O_2_^−•^) generation and elastase release in human neutrophils. The results revealed that, at a concentration of 10 μM, **9** exhibited significant inhibition toward superoxide anion generation and elastase release with IC_50_ values of 10.8 ± 0.38 and 11.0 ± 1.52 μM, respectively.

## 3. Experimental Section

### 3.1. General Experimental Procedures

Optical rotations were measured on a JASCO P-1020 polarimeter (JASCO Corpotation, Tokyo, Japan). IR spectra were recorded on a JASCO FT/IR-4100 infrared spectrophotometer (JASCO Corporation) (Varian Inc., Palo Alto, CA, USA). NMR spectra were recorded on 400 MHz (or 500 MHz) for ^1^H and 100 MHz (or 125 MHz) for ^13^C in CDCl_3_ or C_6_D_6_. LRMS or HRMS were obtained by electrospray ionization (ESI) on a Bucker APEX II mass spectrometer (Bruker, Bremen, Germany). Silica gel (230–400 mesh, Merck, Darmstadt, Germany) was used for column chromatography. Precoated silica gel plates (Merck, Kieselgel 60 F-254, 0.2 mm) were used for analytical thin-layer chromatography (TLC). High-performance liquid chromatography was performed on a Hitachi L-7100 (HPLC) (Hitachi Ltd., Tokyo, Japan) apparatus with a Merck Hibar Si-60 column (250 mm × 21.2 mm, 8 μm) and on a Hitachi L-2455 (HPLC) (Hitachi Ltd., Tokyo, Japan) apparatus with a Sciences Inc. (GL Science, Tokyo, Japan) ODS-3 C18 column (250 mm × 20 mm, 5 μm).

### 3.2. Animal Material

The soft coral *Sinularia flexibilis* was collected by scuba diving off the coast of Liuqiu, Taiwan, in October 2011, at a depth of 10–15 m, and stored in the freezer until extraction. A voucher specimen was deposited in the Department of Marine Biotechnology and Resource, National Sun Yet-sen University.

### 3.3. Extraction and Separation

Sliced bodies of *S. flexibilis* were exhaustively extracted with EtOAc (2 L × 5). The EtOAc extract (40.0 g) was chromatographed over silica gel by column chromatography using hexane, EtOAc–hexane (1:100 and gradually increasing the proportion of EtOAc to 10:1), EtOAc, and then Me_2_CO–EtOAc (1:100 and gradually increasing the proportion of Me_2_CO to 10:1), and subsequently Me_2_CO as eluents to yield 26 fractions. Fraction 16, eluting with hexane–EtOAc (3:1), was further applied on a silica gel column (240 g) using hexane–EtOAc (8:1) to yield five subfractions (A–E). Subfraction 16-C was purified by normal-phase HPLC using hexane–Me_2_CO (8:1) to afford **1** (3.4 mg). Fraction 18, eluting with hexane-EtOAc (1:1), was further purified by RP-18 silica gel and using a mixture of MeOH-H_2_O (1.5:1) to yield seven subfractions (A–G). Subfraction 18-B was purified by reversed-phase HPLC using MeCN-H_2_O (1:2) to afford **3** (1.0 mg). Subfraction 18-D was purified over silica gel column (50 g) using hexane–EtOAc (2:1) to obtain **4** (3.2 mg). Fraction 19, eluting with hexane–EtOAc (1:2), was chromatographed on silica gel (240 g) using hexane–EtOAc (5:1) to yield six subfractions (A–F). Subfraction 19-B was purified on RP-18 silica gel using MeOH–H_2_O (1:1) and subsequently by RP-HPLC with MeOH–H_2_O (1.5:1) to afford **7** (280.4 mg) and **14** (2.9 mg). Subfraction 19-E was purified by RP-HPLC with MeCN–H_2_O (2:1) to yield **2** (94.6 mg), **8** (84.5 mg), and **9** (11.2 mg). Fraction 20, eluting with hexane–EtOAc (2:1), was further purified over silica gel column (200 g) and eluted with hexane–EtOAc (2:1) to yield five subfractions (A–E). Compounds **5** (2.1 mg) and **10** (123.7 mg) were obtained from subfraction 20-C using NP-HPLC (hexane–Me_2_CO, 4:1). Subfraction 20-E was isolated repeatedly over silica gel column (50 g) using hexane–Me_2_CO (3:1) as eluent, followed by RP-HPLC (MeCN–H_2_O, 1:1.5) to afford **6** (4.8 mg), **11** (9.5 mg), **12** (5.1 mg), and **13** (1.0 mg).

Flexibilisin D (**1**): colorless oil; ${\left[\alpha \right]}_{D}^{19}$ −245 (*c* 0.85, CHCl_3_); IR (KBr) v_max_ 2933, 1716, 1647, 1453, 1244, 1149, and 1074 cm^−1^; ^1^H and ^13^C NMR data, see Table 1; ESIMS *m/z* 371 [M + Na]^+^; HRESIMS *m/z* 371.2196 [M + Na]^+^ (calcd. for C_21_H_32_O_4_Na, 371.2198).

Flexibilisin E (**2**): colorless oil; ${\left[\alpha \right]}_{D}^{25}$ +26 (*c* 0.57, CHCl_3_); IR (KBr) v_max_ 3481, 2927, 1713, 1627, 1460, 1438, 1387, 1252, 1159, 946, 816, and 755 cm^−1^; ^1^H and ^13^C NMR data, see Table 1; ESIMS *m/z* 387 [M + Na]^+^; HRESIMS *m/z* 387.2145 [M + Na]^+^ (calcd. for C_21_H_32_O_5_Na, 387.2147).

Secoflexibilisolide A (**3**): colorless oil; ${\left[\alpha \right]}_{D}^{22}$ −580 (*c* 0.28, CHCl_3_); IR (KBr) v_max_ 3450, 2924, 1766, 1672, 1627, 1380, 1232, 1175, 1101, 1078, 1030, and 1026 cm^−1^; ^1^H and ^13^C NMR data, see Table 2; ESIMS *m/z* 371 [M + Na]^+^; HRESIMS *m/z* 371.1831 [M + Na]^+^ (calcd. for C_20_H_28_O_5_Na, 371.1829).

Secoflexibilisolide B (**4**): colorless oil; ${\left[\alpha \right]}_{D}^{19}$ −56 (*c* 0.91, CHCl_3_); IR (KBr) v_max_ 3501, 2924, 1705, 1647, 1515, 1267, and 1153 cm^−1^; ^1^H and ^13^C NMR data, see Table 3; ESIMS *m/z* 387 [M + Na]^+^; HRESIMS *m/z* 387.1779 [M + Na]^+^ (calcd. for C_20_H_28_O_6_Na, 387.1778).

Flexibilisolide H (**5**): white powder; ${\left[\alpha \right]}_{D}^{19}$ −12 (*c* 0.60, CHCl_3_); IR (KBr) v_max_ 3391, 2934, 1739, 1711, 1621, 1239, 1236, 1141, 1065, and 1048 cm^−1^; ^1^H and ^13^C NMR data, see Table 3; ESIMS *m/z* 431 [M + Na]^+^; HRESIMS *m/z* 431.2047 [M + Na]^+^ (calcd. for C_22_H_32_O_7_Na, 431.2046).

### 3.4. Alkaline Hydrolysis of **7**

Compound **7** (3.5 mg) was dissolved in 1 N methanolic NaOH solution (1 mL), and the mixture was stirred at 0 °C for 12 h. The reaction mixture was neutralized with 0.1 N HCl (*aq*). After evaporation of the solvent, the residue was extracted with CHCl_3_, and subsequently purified by HPLC using MeOH–H_2_O (3:1) as eluent to yield a methyl ester (1.1 mg, 31.4%; ${\left[\alpha \right]}_{D}^{25}$ +48 (*c* 0.28, CHCl_3_; ^1^H and ^13^C NMR spectra, Supplementary materials, Figures S4 and S5), which was identified as **2**.

### 3.5. Cytotoxicity Testing

Cell lines were purchased from the American Type Cultural Collection (ATCC). Cytotoxicity assay of **1**, **2**, and **4**–**14** were performed using Alamar Blue Assay [32,33].

### 3.6. In Vitro Anti-Inflammatory Assay

Human neutrophils were obtained from whole blood using dextran sedimentation and Ficoll centrifugation. Measurements of superoxide anion generation and elastase release were performed according to previously described procedures [34,35]. Idelalisib was used as a positive control, of which the IC_50_ values for inhibition of superoxide anion generation and elastase release were 0.07 ± 0.01 and 0.28 ± 0.09 μM, respectively.

## 4. Conclusions

Five new diterpenoids and nine known compounds were isolated and characterized from the marine soft coral *Sinularia flexibilis*. The previously unreported **3**, containing a bicyclo[4.3.0]nonane ring system, was proposed be derived from flexibilisolide D. Compounds **7** and **8** showed selective cytotoxicity toward P388 cancer cell line, while **8** also exhibited significant cytotoxicity toward HT-29 cancer cells. Compound **9** displayed weaker cytotoxicity than **7** and **8**, but displayed potent inhibitory activities for superoxide anion generation and elastase release in (fMLF-CB)-induced human neutrophils.

## Figures and Tables

**Table 1:** ^1^H and ^13^C NMR spectroscopic data of **1** and **2**.

Position	1		2	
	*δ*_H_^a^ (*J* in Hz) ^c^	*δ*_C_^b^ (mult.) ^d^	*δ*_H_ (*J* in Hz)	*δ*_C_ (mult.)
1	2.85, m	35.2 (CH)	2.79, m	36.5 (CH)
2	2.05, m; 1.36, m	31.5 (CH_2_)	1.98, m; 1.40, m	32.5 (CH_2_)
3	2.81, dd (10.0, 4.0)	60.1 (CH)	2.77, dd; (9.6, 4.4)	59.4 (CH)
4		60.2 (C)		60.7 (C)
5	2.00, m; 1.59, m	36.9 (CH_2_)	1.99, m; 1.54, m	36.8 (CH_2_)
6	2.12, m; 2.08, m	22.2 (CH_2_)	2.10, m; 2.00, m	22.9 (CH_2_)
7	5.18, t (5.6)	126.5 (CH)	5.11, t (5.2)	126.4 (CH)
8		134.2 (C)		134.6 (C)
9	2.26, m; 2.09, m	36.0 (CH_2_)	2.55, m, 2.22, m	31.6 (CH_2_)
10	2.09, m; 1.40, m	24.6 (CH_2_	2.71, ddd (10.8, 8.0, 2.8)	34.3 (CH_2_)
			2.65, ddd (10.8, 8.0, 2.8)	
11	2.65, dd (10.0, 3.2)	61.9 (CH)		213.6 (C)
12		60.8 (C)		78.8 (C)
13	1.70, m; 1.20, m	33.0 (CH_2_)	1.74, ddd (12.8, 5.6. 2.0) 1.48, m	36.1 (CH_2_)
14	1.70, m; 1.58, m	27.4 (CH_2_)	1.54, m, 1.37, m	25.2 (CH_2_)
15		142.7 (C)		142.2 (C)
16		167.3 (C)		167.4 (C)
17	6.26, s; 5.48, s	124.4 (CH_2_)	6.31, s, 5.49, s	124.4 (CH_2_)
18	1.29, s	17.8 (CH_3_)	1.27, s	18.2 (CH_3_)
19	1.65, s	15.3 (CH_3_)	1.66, s	17.1 (CH_3_)
20	1.23, s	17.0 (CH_3_)	1.34, s	25.7 (CH_3_)
16-OMe	3.76, s	52.0 (CH_3_)	3.76, s	52.0 (CH_3_)

^a^ Spectra recorded at 400 MHz in CDCl_3_. ^b^ Spectra recorded at 100 MHz in CDCl_3_. ^c^ *J* values (in Hz) in parentheses. ^d^ Attached protons were deduced by DEPT experiments.

**Table 2:** ^1^H, ^13^C NMR data, COSY, and HMBC correlations of **3**.

Position	*δ*_H_^a^ (*J* in Hz) ^c^	*δ*_C_^b^ (mult.) ^d^	COSY	HMBC
1	2.13, m	34.5 (CH)	H-2, 14	C-3, 16
2	1.78, m; 1.38, m	30.7 (CH_2_)	H-1, 3	C-1, 3, 14, 15
3	2.74, dd (7.5, 6.5)	61.9 (CH)	H-2	C-2
4		58.8 (C)		
5	2.44, d (7.5)	41.2 (CH_2_)	H-6	C-3, 4, 6, 7, 18
6	6.73, m	142.2 (CH)	H-5, 7	C-4, 5, 7, 8
7	6.12, d (16.0)	134.0 (CH)	H-6	C-8
8		198.3 (C)		
9	1.82, m	20.8 (CH_2_)	H-10, 17	C-15
10	2.05, m; 1.68, m	34.1 (CH_2_)	H-9	C-9, 11, 12
11		85.3 (C)		
12		84.7 (C)		
13	2.10, m; 1.72, m	30.4 (CH_2_)		C-11, 12
14	1.96, m; 1.22, m	25.8 (CH_2_)		C-12, 15
15		60.9 (C)		
16		177.6 (C)		
17	2.26, m; 1.66, m	28.4 (CH_2_)	H-9	C-1, 15, 16
18	1.28, s	17.2 (CH_3_)		C-3, 4, 5
19	2.26, s	27.0 (CH_3_)		C-7, 8
20	1.39, s	18.7 (CH_3_)		C-11, 12, 13

^a^ Spectra recorded at 500 MHz in CDCl_3_. ^b^ Spectra recorded at 125 MHz in CDCl_3_. ^c^ *J* values (in Hz) in parentheses. ^d^ Attached protons were deduced by DEPT experiments.

**Table 3:** ^1^H and ^13^C NMR data of compounds **4** and **5.**

Position	4		5	
	*δ*_H_^a^ (*J* in Hz) ^e^	*δ*_C_^b^ (mult.) ^f^	*δ*_H_^c^ (*J* in Hz)	*δ*_C_^d^ (mult.)
1	2.02, m	35.9 (CH)	2.92, m	34.6 (CH)
2	1.07, m; 1.02, m	28.2 (CH_2_)	2.14, m; 1.48, m	33.3 (CH_2_)
3	3.64, dd (11.6)	83.0 (CH)	3.07, dd (10.0, 4.0)	61.7 (CH)
4		72.5 (C)		59.6 (C)
5	2.14, m; 1.97, m	41.3 (CH_2_)	2.12, m; 1.48, m	34.3 (CH_2_)
6	5.66, m	124.1 (CH)	1.94, m; 1.91, m	25.5 (CH_2_)
7	5.20, dd (15.6, 6.8)	136.9 (CH)	4.53, d (10.0)	81.2 (CH)
8		83.9 (C)		145.8 (C)
9	1.44, m; 1.22, m	33.9 (CH_2_)	2.59, m; 2.23, m	29.7 (CH_2_)
10	2.05, m; 1.94, m	28.7 (CH_2_)	1.89, m; 1.78, m	26.5 (CH_2_)
11		175.4 (C)	5.60, dd (12.8, 2.4)	72.9 (CH)
12		205.6 (C)		87.1 (C)
13	1.76, m; 1.68, m	39.4 (CH_2_)	2.03, m; 1.92, m	32.8 (CH_2_)
14	1.62, m; 1.32, m	28.0 (CH_2_)	2.23, m; 1.38, m	30.1 (CH_2_)
15		139.5 (C)		143.4 (C)
16		165.3 (C)		168.1 (C)
17	6.40, s; 5.12, s	126.0 (CH_2_)	6.30, s; 5.50, s	125.0 (CH_2_)
18	1.00 s	22.4 (CH_3_)	1.40 s	24.0 (CH_3_)
19	1.04 s	26.3 (CH_3_)	5.09 s; 5.06 s	115.0 (CH_2_)
20	1.64 s	29.4 (CH_3_)	1.38 s	15.5 (CH_3_)
OAc			2.09 s	20.9 (CH_3_)
				170.6 (C)

^a^ Spectra recorded at 400 MHz in C_6_D_6_. ^b^ Spectra recorded at 100 MHz in C_6_D_6_. ^c^ Spectra recorded at 400 MHz in CDCl_3_. ^d^ Spectra recorded at 100 MHz in CDCl_3_. ^e^ J values (in Hz) in parentheses. ^f^ Attached protons were deduced by DEPT experiments.

**Table 4:** The cytotoxic activity of **7**–**9** and **11** ^a^.

Compound	IC_50_ (μM)		
	P-388 ^b^	K-562 ^c^	HT-29 ^d^
**7**	9.3	23.4	15.9
**8**	6.9	12.2	9.6
**9**	16.0	26.7	(–)
**11**	(–) ^e^	21.7	27.1
Doxorubicin hydrochloride ^f^	0.3	1.0	0.9

^a^ Compounds **1**, **2**, **4**–**6**, **10**, and **12**–**14** were inactive toward the three cancer cell lines with IC_50_ > 40 μM. ^b^ P-388: murine leukemia. ^c^ K-562: human erythromyeloblastoid leukemia. ^d^ HT-29: human colon adenocarcinoma. ^e^ (–): IC_50_ > 40 μM. ^f^ Positive control.

## Supplementary Materials

^1^H and ^13^C spectra of compounds **1**–**14** and the hydrolyzed product of **7** are available online at http://www.mdpi.com/1660-3397/16/8/278/s1. Figure S1: ^1^H NMR spectrum of compound **1** in CDCl_3_, Figure S2: ^13^C NMR spectrum of compound **1** in CDCl_3_, Figure S3: ^1^H NMR spectrum of compound **2** in CDCl_3_, Figure S4: ^1^H NMR spectra of compound **2** and hydrolyzed product of **7** in CDCl_3_, Figure S5: ^13^C NMR spectra of compound **2** and hydrolyzed product of **7** in CDCl_3_, Figure S6: ^13^C NMR spectrum of compound **2** in CDCl_3_, Figure S7: ^1^H NMR spectrum of compound **3** in CDCl_3_, Figure S8: ^1^H NMR spectrum of compound **3** in pyridine-*d_5_*, Figure S9: ^13^C NMR spectrum of compound **3** in CDCl_3_, Figure S10: ^1^H–^1^H COSY spectrum of **3** in pyridine-*d_5_*, Figure S11: ^1^H NMR spectrum of compound **4** in C_6_D_6_, Figure S12: ^13^C NMR spectrum of compound **4** in C_6_D_6_, Figure S13: ^1^H NMR spectrum of compound **5** in CDCl_3_, Figure S14: ^13^C NMR spectrum of compound **5** in CDCl_3_, Figure S15: ^1^H NMR spectrum of compound **6** in CDCl_3_, Figure S16: ^13^C NMR spectrum of compound **6** in CDCl_3_, Figure S17: ^1^H NMR spectrum of compound **7** in CDCl_3_, Figure S18: ^13^C NMR spectrum of compound **7** in CDCl_3_, Figure S19: ^1^H NMR spectrum of compound **8** in CDCl_3_, Figure S20: ^13^C NMR spectrum of compound **8** in CDCl_3_, Figure S21: ^1^H NMR spectrum of compound **9** in CDCl_3_, Figure S22: ^13^C NMR spectrum of compound **9** in CDCl_3_, Figure S23: ^1^H NMR spectrum of compound **10** in CDCl_3_, Figure S24: ^13^C NMR spectrum of compound **10** in CDCl_3_, Figure S25: ^1^H NMR spectrum of compound **11** in CDCl_3_, Figure S26: ^13^C NMR spectrum of compound **11** in CDCl_3_, Figure S27: ^1^H NMR spectrum of compound **12** in CDCl_3_, Figure S28: ^13^C NMR spectrum of compound **12** in CDCl_3_, Figure S29: ^1^H NMR spectrum of compound **13** in CDCl_3_, Figure S30: ^13^C NMR spectrum of compound **13** in CDCl_3_, Figure S31: ^1^H NMR spectrum of compound **14** in CDCl_3_, Figure S32: ^13^C NMR spectrum of compound **14** in CDCl_3_.
