# Supplementary material for: Cembranoid-Related Metabolites and Biological Activities from the Soft Coral Sinularia flexibilis
Source: Mar Drugs. 2018 Aug 9;16(8):278. doi: 10.3390/md16080278 (PMC6117644; doi:10.3390/md16080278)
Supplement: Supplementary file 1 [file marinedrugs-16-00278-s001.pdf]

Secondary Metabolites and Biological Activities from the Soft Coral *Sinularia flexibilis*

Chia-Hua Wu[1,†], Chao, Chih-Hua[2,3,†], Tzu-Zin Huang[1,†], Chiung-Yao Huang[1], Tsong-Long Hwang[4], Chang-Feng Dai[5] and Jyh-Horng Sheu[1,6,7,8,*]

[1] Department of Marine Biotechnology and Resources, National Sun Yat-sen University, Kaohsiung 804, Taiwan; cathywu7979@gmail.com(C.-H.W.)slime112229@gmail.com (T.-Z.H.); huangcy@mail.nsysu.edu.tw (C.-Y.H.);

[2] School of Pharmacy, China Medical University, Taichung 404, Taiwan; chchao@mail.cmu.edu.tw

[3] Chinese Medicine Research and Development Center, China Medical University Hospital, Taichung 404, Taiwan; chchao@mail.cmu.edu.tw

[4] Graduate Institute of Natural Products, College of Medicine, Chang Gung University; Research Center for Industry of Human Ecology and Graduate Institute of Health Industry Technology, Chang Gung University of Science and Technology; Department of Anesthesiology, Chang Gung Memorial Hospital, Taoyuan 333, Taiwan; htl@mail.cgu.edu.tw (T.-L.H.)

[5] Institute of Oceanography, National Taiwan University, Taipei 112, Taiwan; corallab@ntu.edu.tw (C.-F.D.)

[6] Institute of Natural Products, Kaohsiung Medical University, Kaohsiung 807, Taiwan

[7] Department of Medical Research, China Medical University Hospital, China Medical University, Taichung 404, Taiwan

[8] Frontier Center for Ocean Science and Technology, National Sun Yat-sen University, Kaohsiung 804, Taiwan

*Correspondence: sheu@mail.nsysu.edu.tw; Tel: +886-7-5252000 (ext. 5030); Fax: +886-7-5255020

[†] These authors contributed equally to this work

*To whom correspondence should be addressed. Tel.: 886-7-5252000 ext. 5030, Fax: 886-7-5255020. E-mail: sheu@mail.nsysu.edu.tw

No	Content	Page
Figure S1.	^{1}H NMR spectrum (500 MHz) of compound **1** in $CDCl_3$.	3
Figure S2.	^{13}C NMR spectrum (100 MHz) of compound **1** in $CDCl_3$.	4
Figure S3.	^{1}H NMR spectrum (500 MHz) of compound **2** in $CDCl_3$.	5
Figure S4.	^{1}H NMR spectrum (400 MHz) of compound **2** and hydrolyzed product of **7** in $CDCl_3$.	6
Figure S5.	^{13}C NMR spectrum (100 MHz) of compound **2** in $CDCl_3$.	7
Figure S6.	^{1}H NMR spectrum (500 MHz) of compound **3** in $CDCl_3$.	8
Figure S7.	^{1}H NMR spectrum (500 MHz) of compound **3** in Pyridine.	9
Figure S8.	^{13}C NMR spectrum (100 MHz) of compound **3** in $CDCl_3$.	10
Figure S9.	^{1}H–^{1}H COSY spectrum of **3** in Pyridine.	11
Figure S10.	^{1}H NMR spectrum (500 MHz) of compound **4** in C_6D_6.	12
Figure S11.	^{13}C NMR spectrum (100 MHz) of compound **4** in C_6D_6.	13
Figure S12.	^{1}H NMR spectrum (500 MHz) of compound **5** in $CDCl_3$.	14
Figure S13.	^{13}C NMR spectrum (100 MHz) of compound **5** in $CDCl_3$.	15

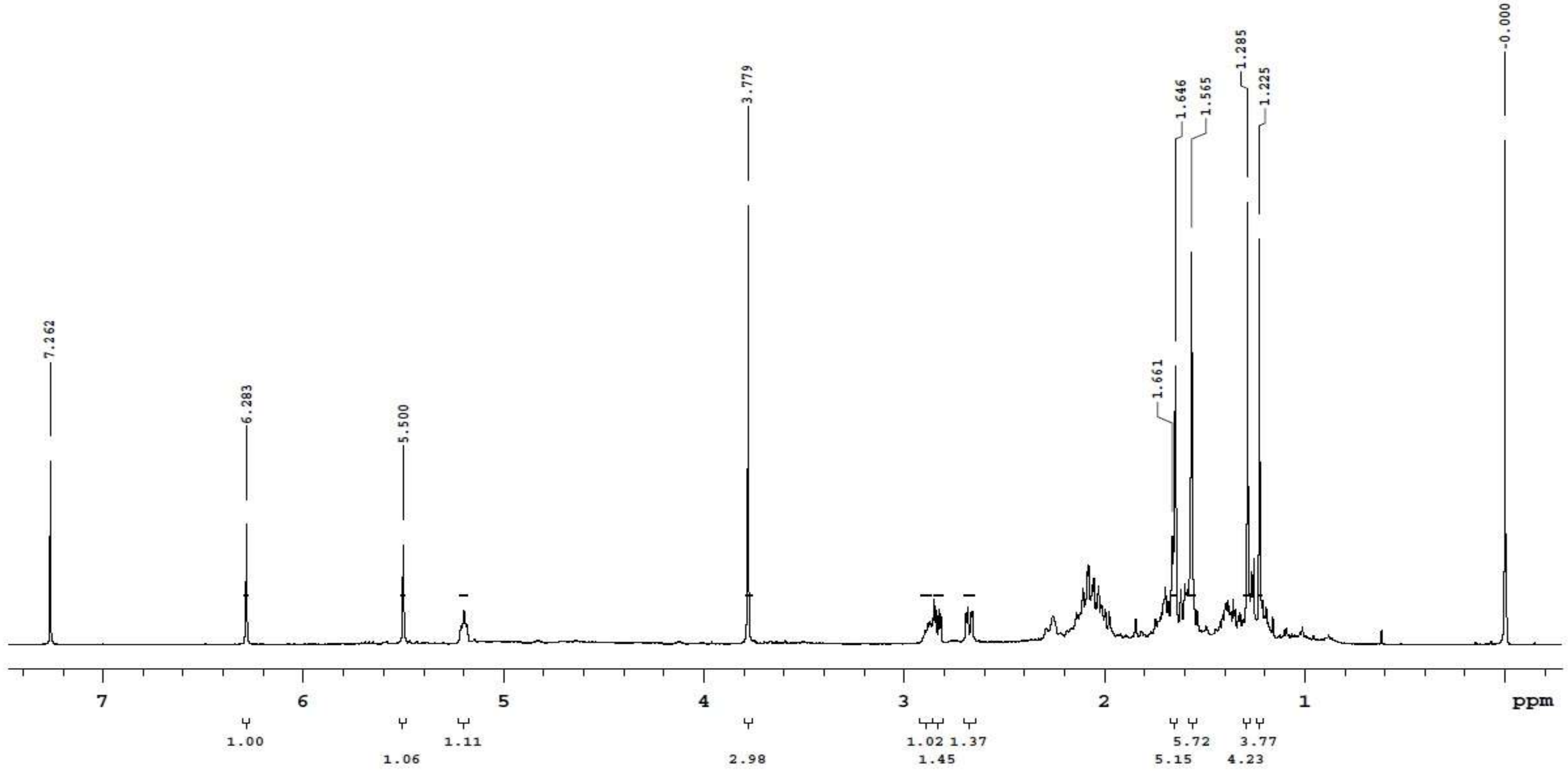

Figure S1. ^{1}H NMR spectrum (500 MHz) of compound **1** in $CDCl_3$.

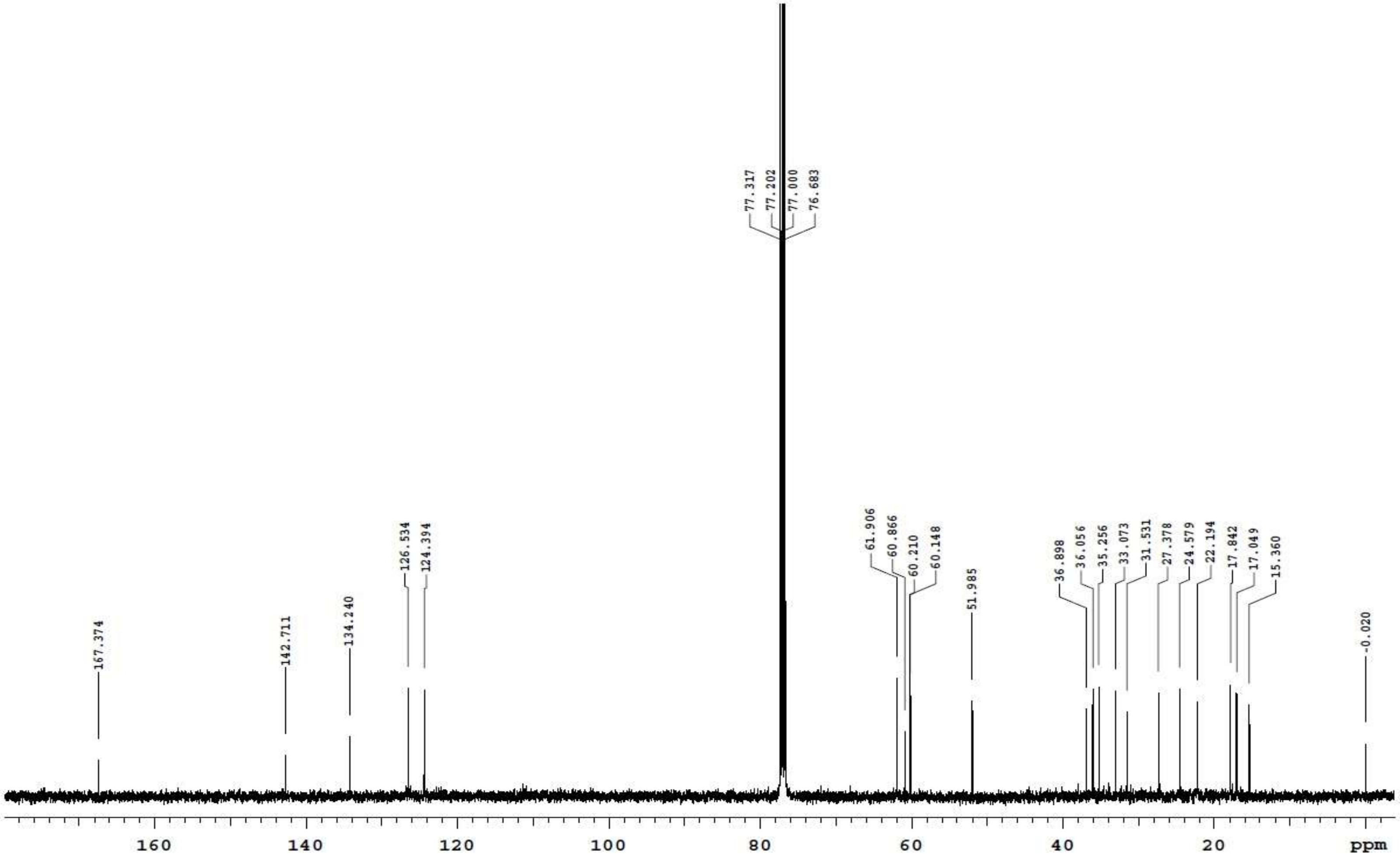

Figure S2. ^{13}C NMR spectrum (100 MHz) of compound **1** in $CDCl_3$.

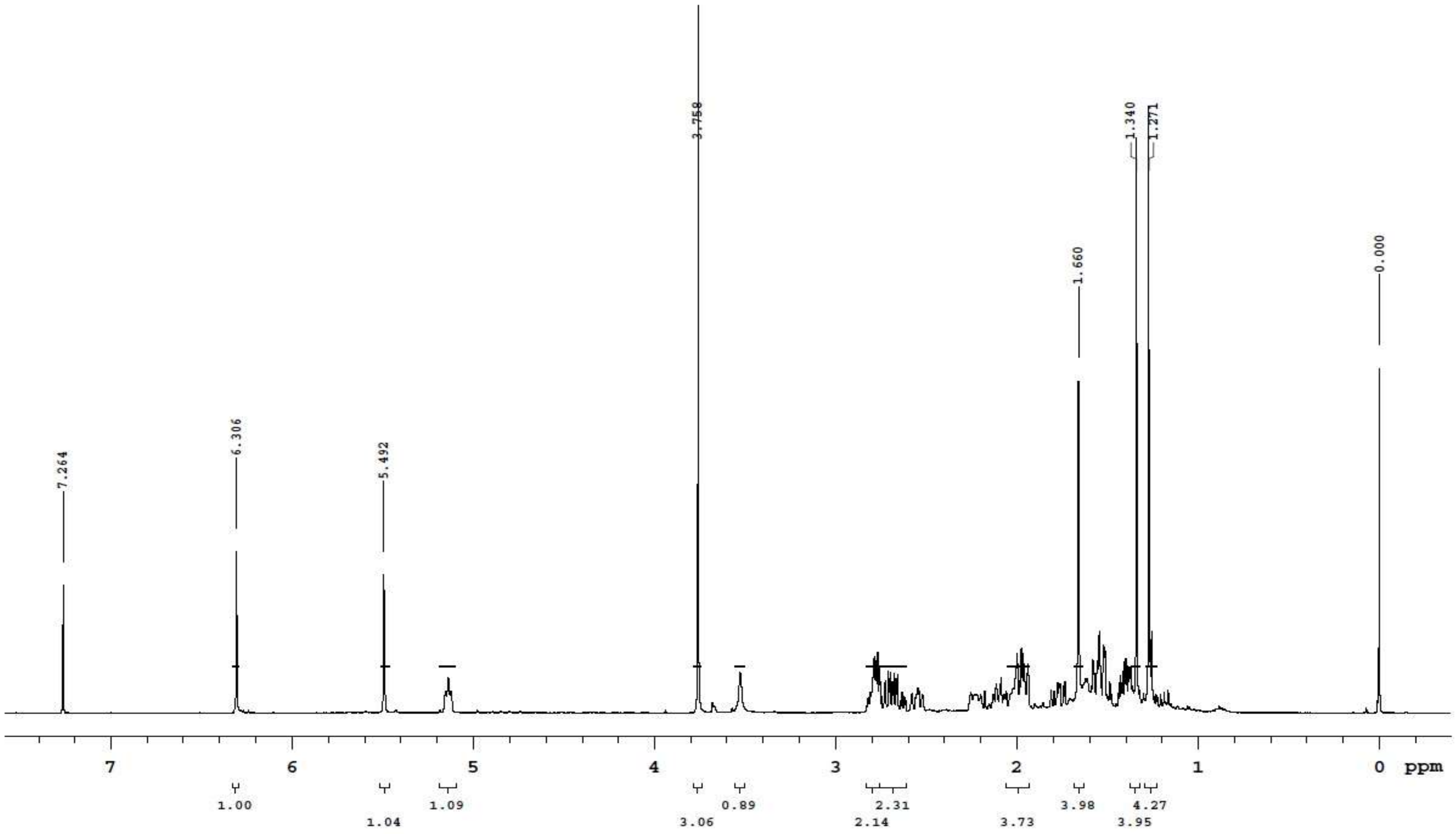

Figure S3. ^{1}H NMR spectrum (500 MHz) of compound **2** in $CDCl_3$.

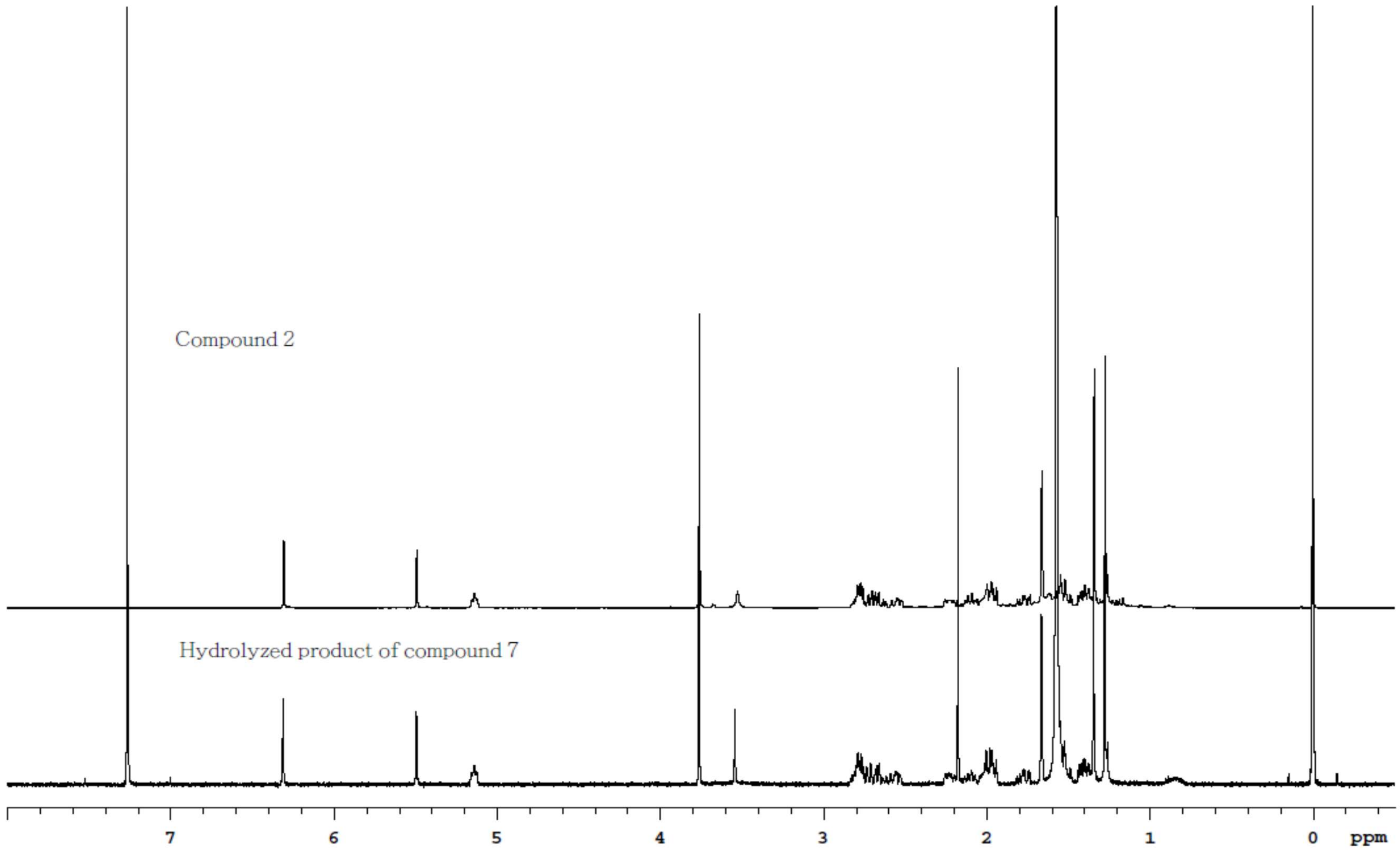

Figure S4. ^{1}H NMR spectrum (400 MHz) of compound **2** and hydrolyzed product of **7** in $CDCl_3$.

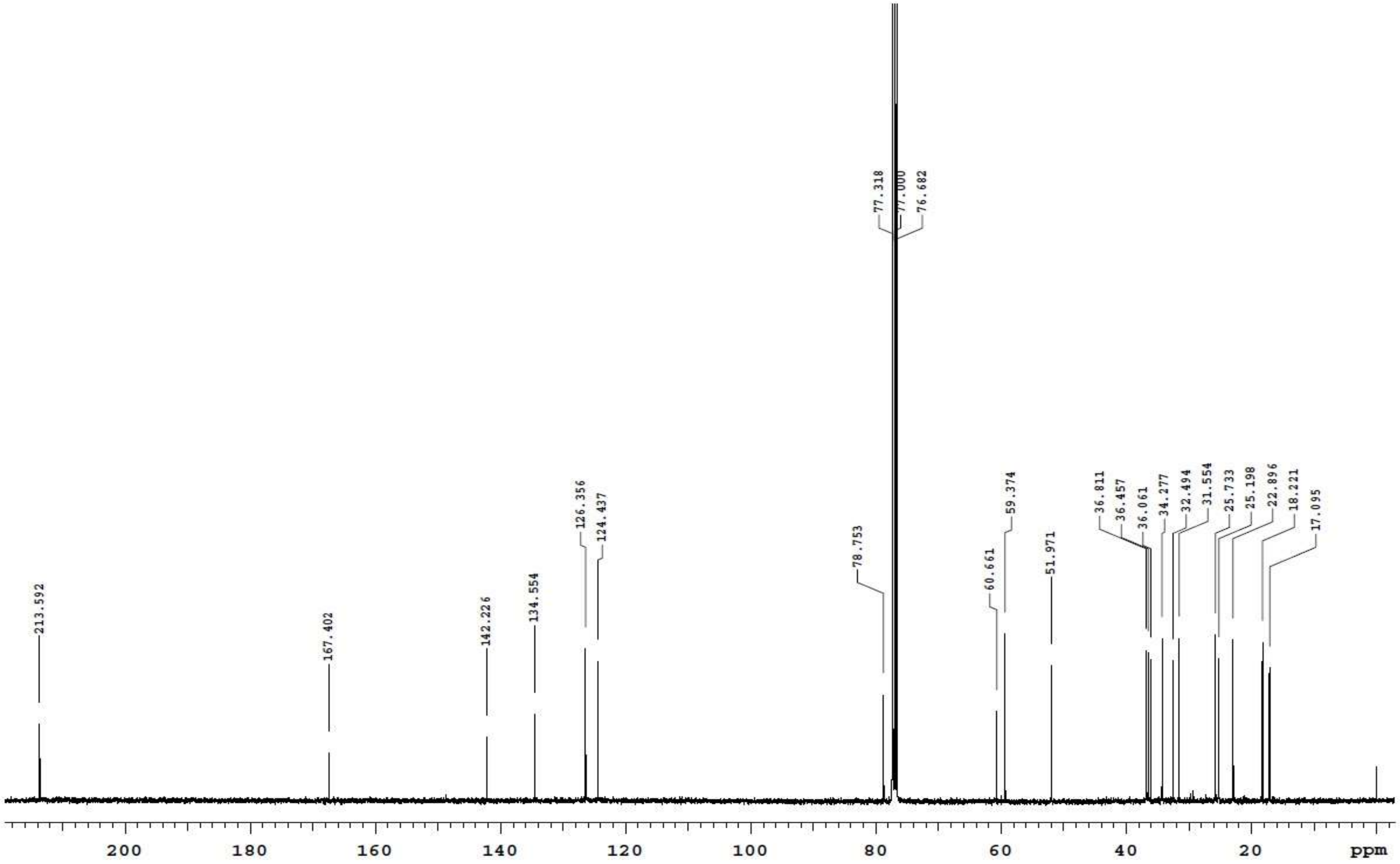

Figure S5. ^{13}C NMR spectrum (100 MHz) of compound **2** in $CDCl_3$.

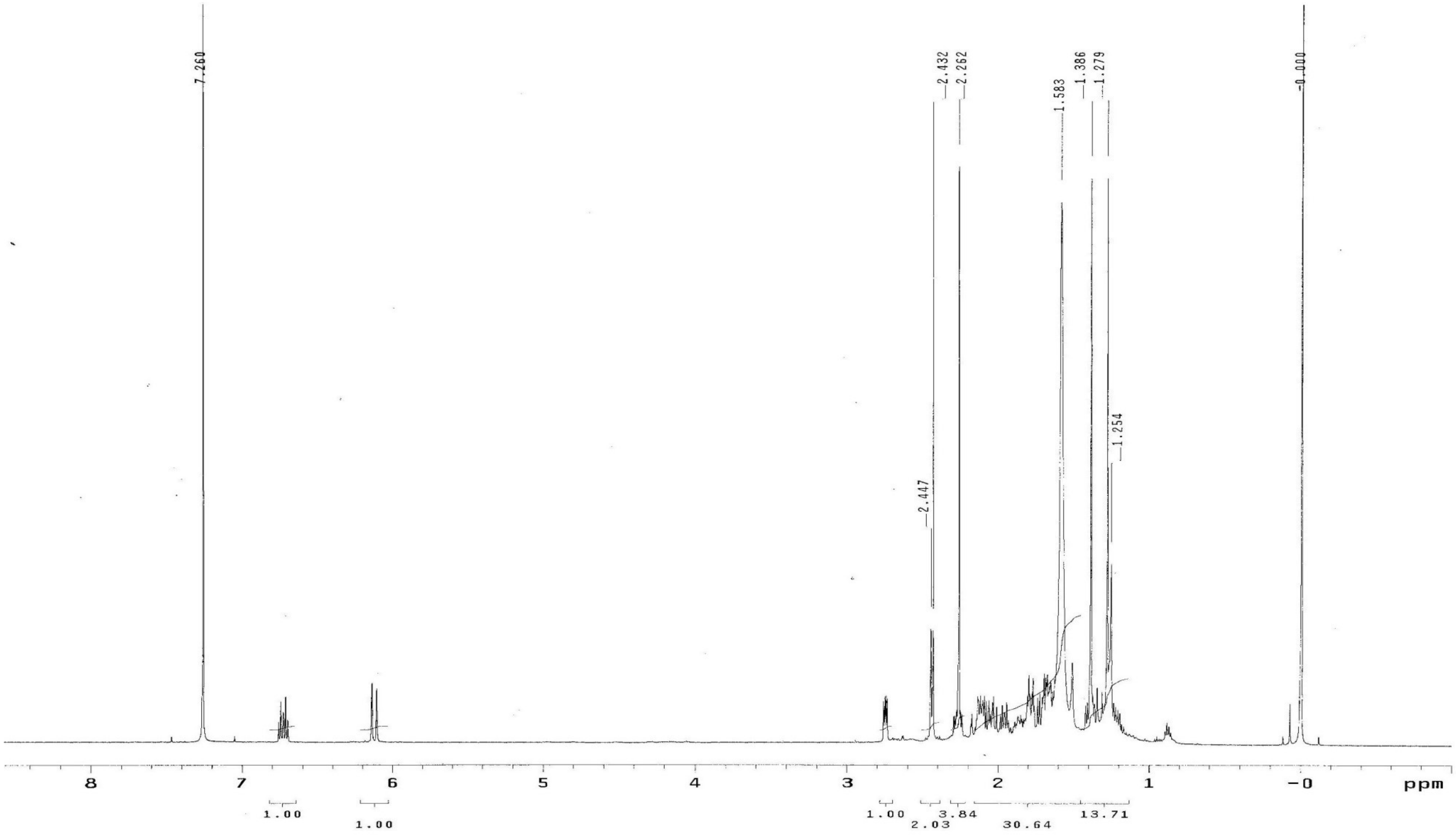

Figure S6. ^{1}H NMR spectrum (500 MHz) of compound **3** in $CDCl_3$.

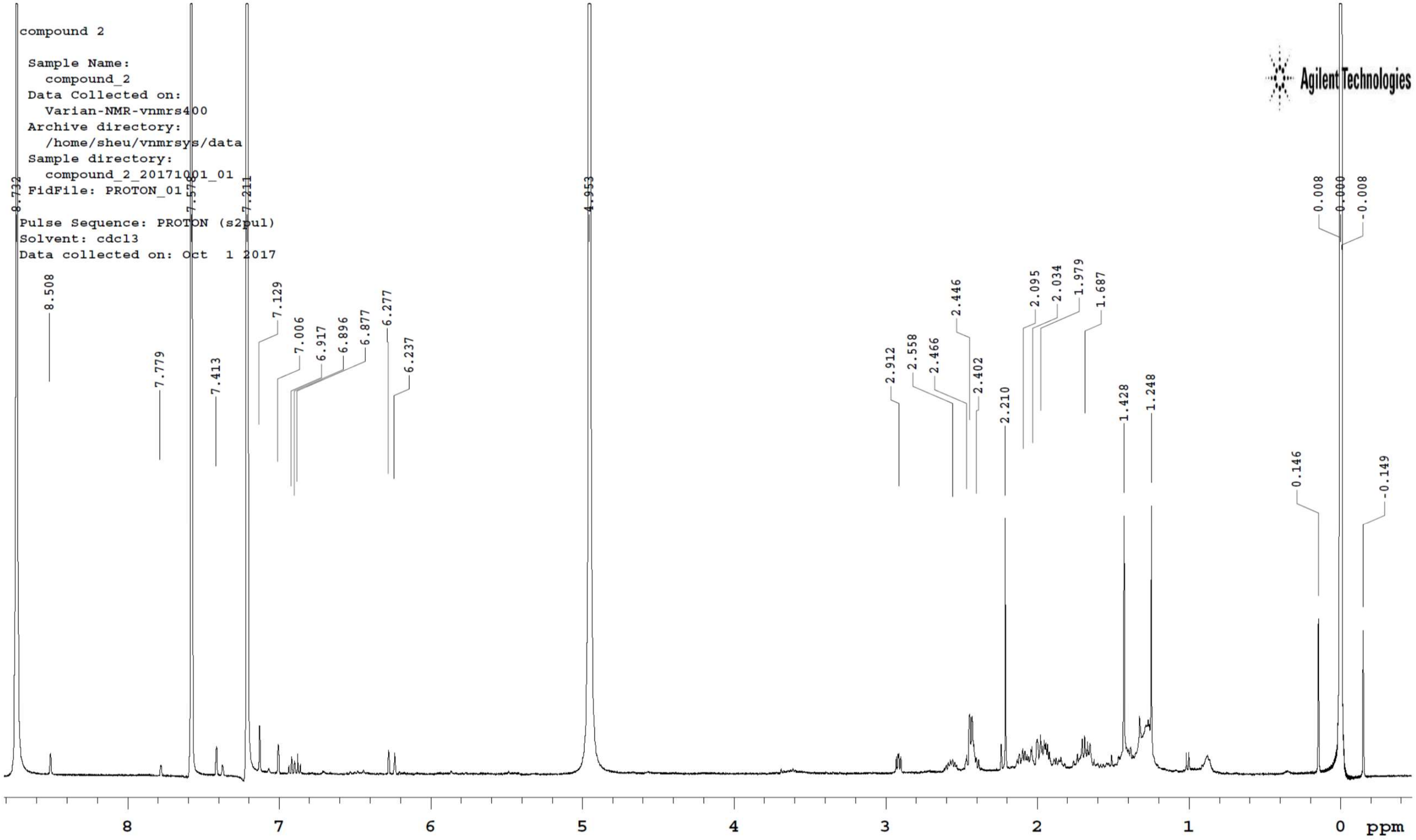

Figure S7. ^{1}H NMR spectrum (500 MHz) of compound **3** in Pyridine.

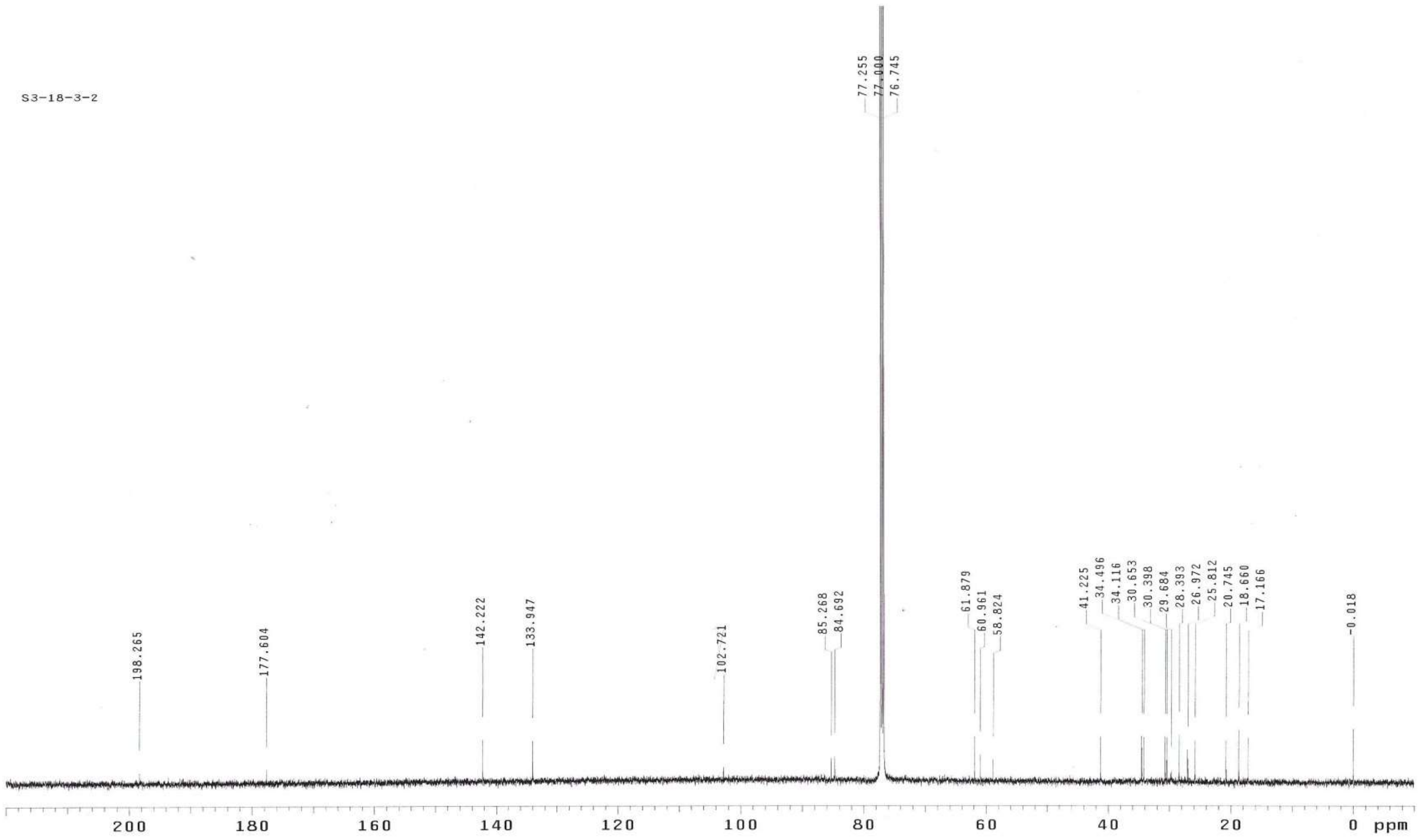

Figure S8. ^{13}C NMR spectrum (100 MHz) of compound **3** in CDCl3.

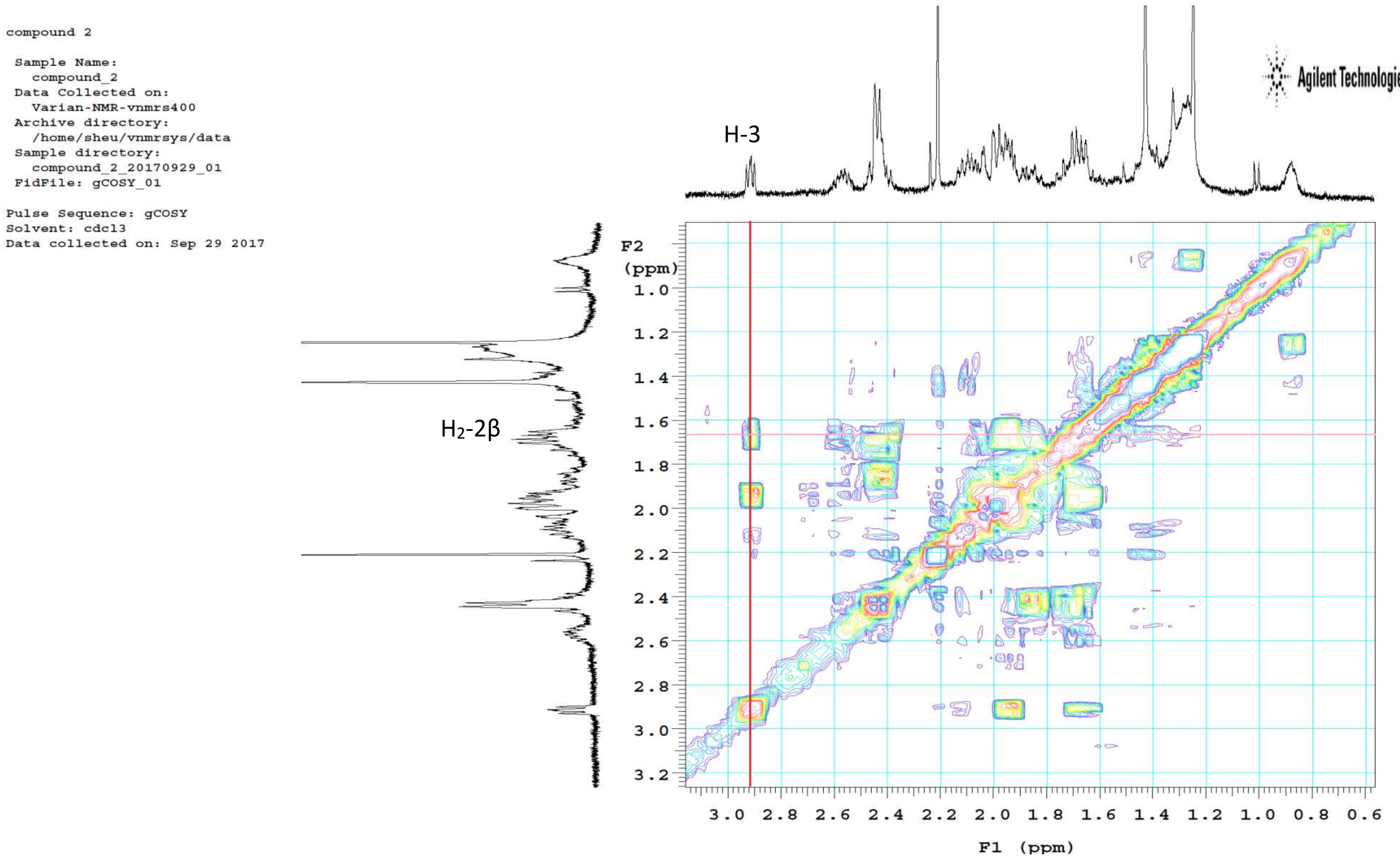

Figure S9. ^1H–^1H COSY spectrum of **3** in Pyridine.

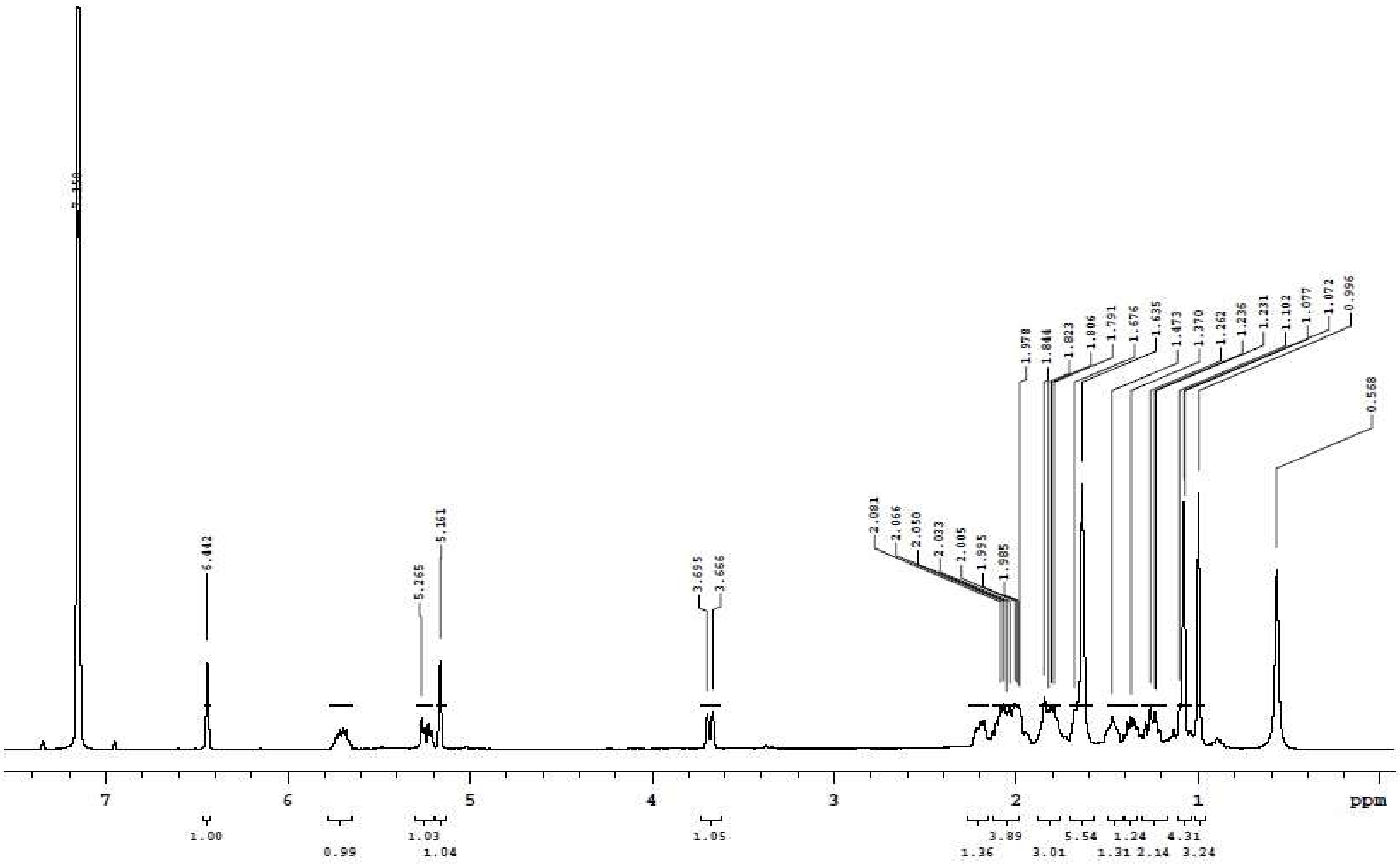

Figure S10. ^{1}H NMR spectrum (500 MHz) of compound **4** in C_6D_6.

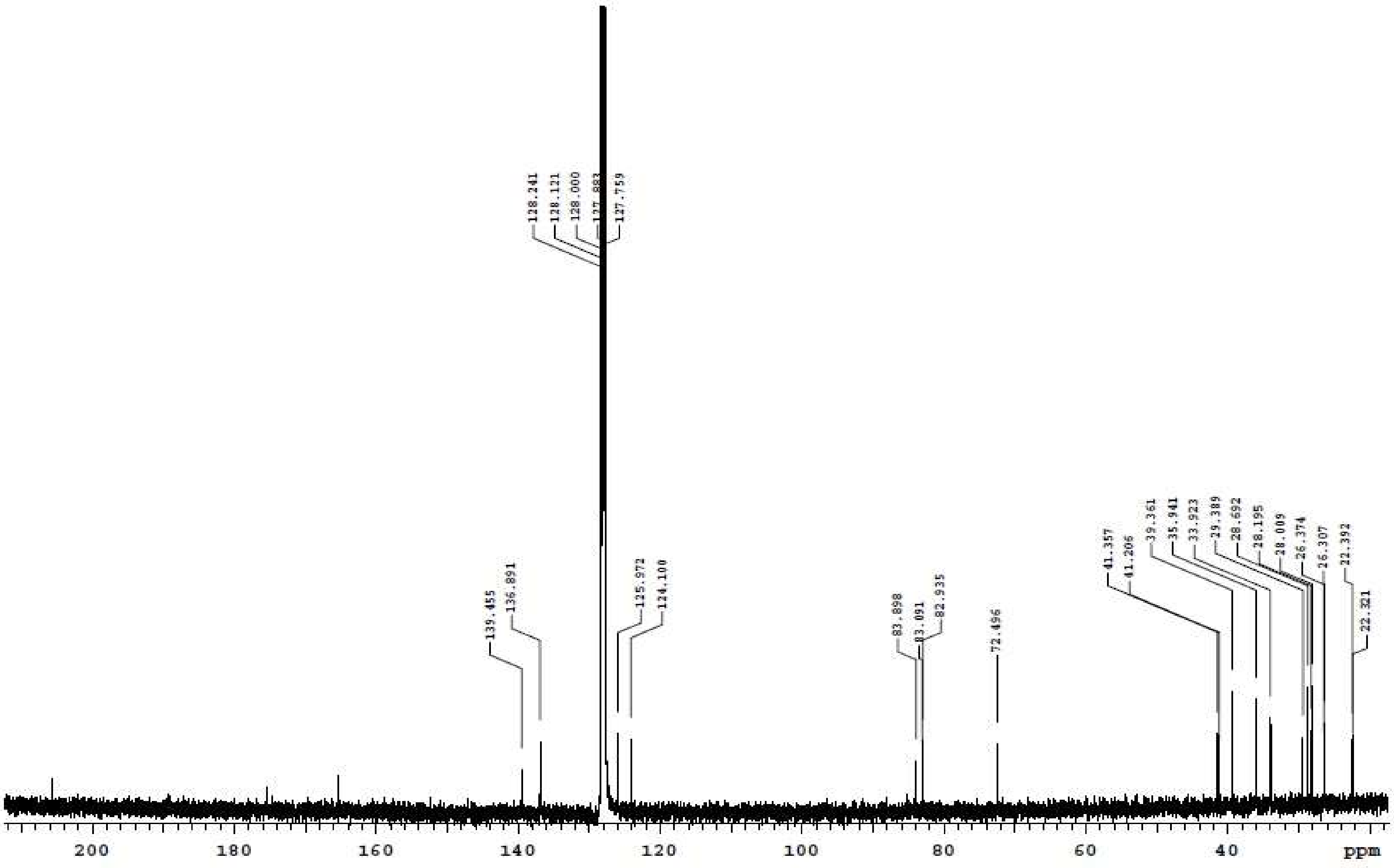

Figure S11. ^{13}C NMR spectrum (100 MHz) of compound **4** in C_6D_6.

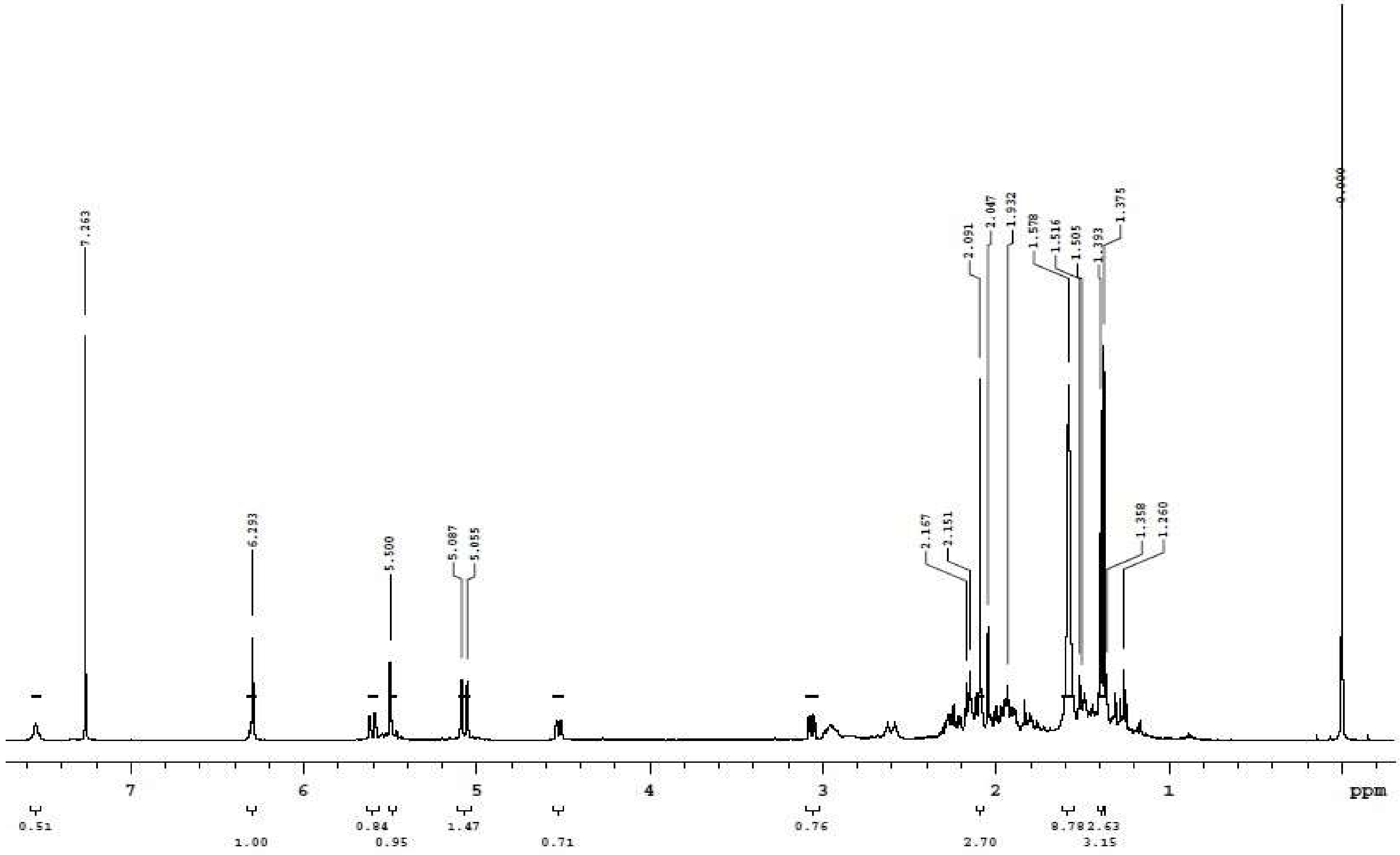

Figure S12. ^{1}H NMR spectrum (500 MHz) of compound **5** in $CDCl_3$.

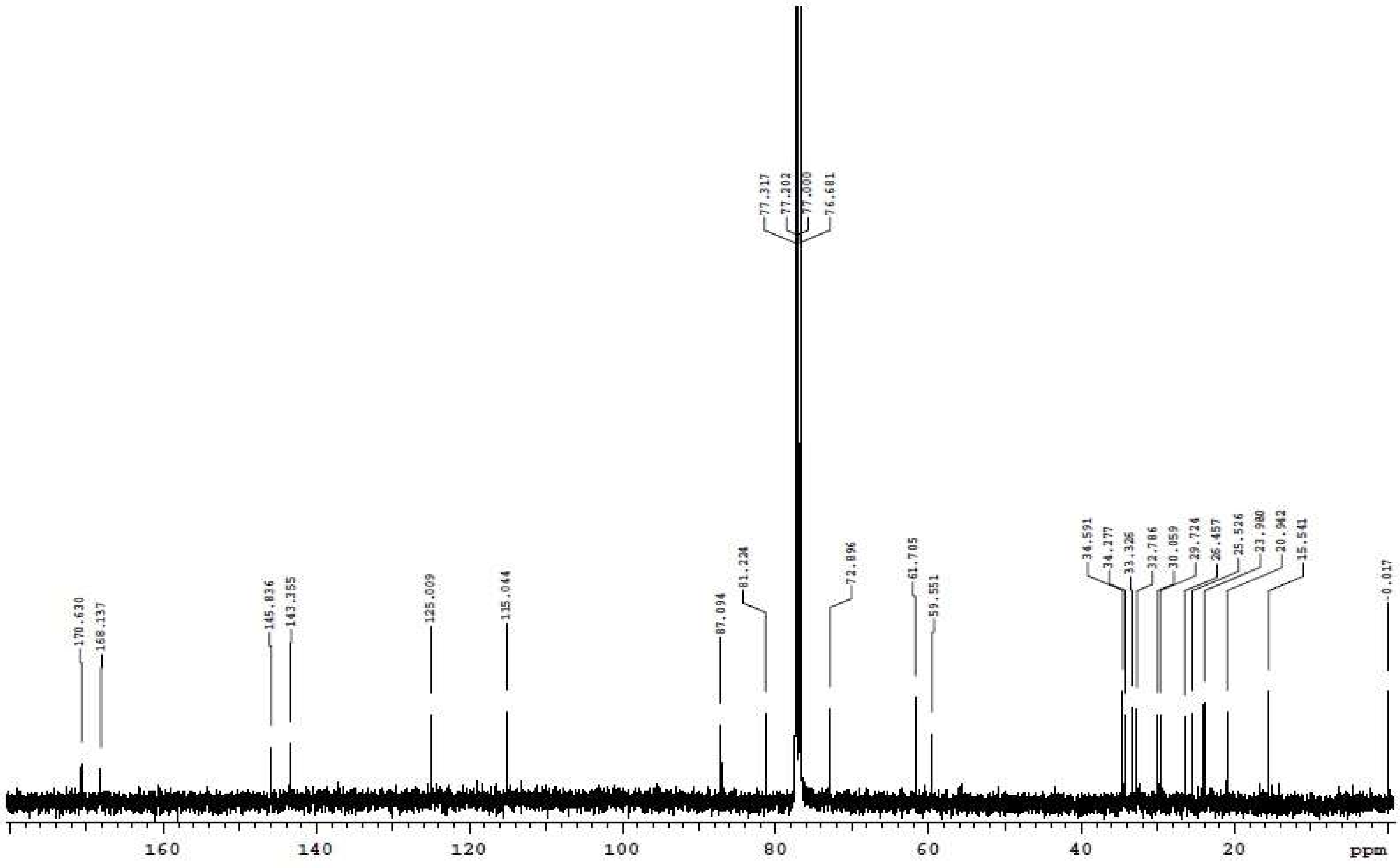

Figure S13. ^{13}C NMR spectrum (100 MHz) of compound **5** in $CDCl_3$.
